# Supplementary material for: The Japanese version of the Generalized Problematic Internet Use Scale 2 (GPIUS2): Psychometric evaluation and analysis of the theoretical model
Source: PLoS One. 2022 Nov 28;17(11):e0273895. doi: 10.1371/journal.pone.0273895 (PMC9704612; doi:10.1371/journal.pone.0273895)
Supplement: S1 Table — (DOCX) [file pone.0273895.s001.docx]

**S1 Table.** Partial Correlations coefficients between the GPIUS2 scales and IAT, self-esteem, depression, and impulsivity with age as a covariate based on listwise deletion (*n* = 128).

**p* < .05; ***p* < .01; ****p* < .001
