# Supplementary material for: The Japanese version of the Generalized Problematic Internet Use Scale 2 (GPIUS2): Psychometric evaluation and analysis of the theoretical model
Source: PLoS One. 2022 Nov 28;17(11):e0273895. doi: 10.1371/journal.pone.0273895 (PMC9704612; doi:10.1371/journal.pone.0273895)
Supplement: Attachment — Submitted filename: 220812 response to reviewers.docx [file pone.0273895.s003.docx]

Response to academic editor

*Comment 1*: Please consider minor typo errors.

*Authors’ reply*: We have corrected typing errors in the new manuscript.

*Comment 2*: Please remove the suggested reference n 4 (line 499)

*Authors’ reply*: We removed reference number 4 in the old manuscript. Additionally, subsequent literature numbers have been changed.

Response to Journal requirements

*Comment 1*: Please review your reference list to ensure that it is complete and correct. If you have cited papers that have been retracted, please include the rationale for doing so in the manuscript text, or remove these references and replace them with relevant current references. Any changes to the reference list should be mentioned in the rebuttal letter that accompanies your revised manuscript. If you need to cite a retracted article, indicate the article’s retracted status in the References list and also include a citation and full reference for the retraction notice.

*Authors’ reply*: After reviewing and revising our manuscript (reference number 4 in the old manuscript was removed), we have confirmed that our reference list is complete and correct.
